# The osteocyte and its osteoclastogenic potential

**DOI:** 10.3389/fendo.2023.1121727

**Published:** 2023-05-24

**Authors:** Aseel Marahleh, Hideki Kitaura, Fumitoshi Ohori, Takahiro Noguchi, Itaru Mizoguchi

**Affiliations:** ^1^ Frontier Research Institute for Interdisciplinary Sciences (FRIS), Tohoku University, Sendai, Japan; ^2^ Division of Orthodontics and Dentofacial Orthopedics, Graduate School of Dentistry, Tohoku University, Sendai, Japan

**Keywords:** osteocyte, osteoclast, lacunocanalicular network, anosteocytic bone, RANKL

## Abstract

The skeleton is an organ of dual functionality; on the one hand, it provides protection and structural competence. On the other hand, it participates extensively in coordinating homeostasis globally given that it is a mineral and hormonal reservoir. Bone is the only tissue in the body that goes through strategically consistent bouts of bone resorption to ensure its integrity and organismal survival in a temporally and spatially coordinated process, known as bone remodeling. Bone remodeling is directly enacted by three skeletal cell types, osteoclasts, osteoblasts, and osteocytes; these cells represent the acting force in a basic multicellular unit and ensure bone health maintenance. The osteocyte is an excellent mechanosensory cell and has been positioned as the choreographer of bone remodeling. It is, therefore, not surprising that a holistic grasp of the osteocyte entity in the bone is warranted. This review discusses osteocytogenesis and associated molecular and morphological changes and describes the osteocytic lacunocanalicular network (LCN) and its organization. We highlight new knowledge obtained from transcriptomic analyses of osteocytes and discuss the regulatory role of osteocytes in promoting osteoclastogenesis with an emphasis on the case of osteoclastogenesis in anosteocytic bones. We arrive at the conclusion that osteocytes exhibit several redundant means through which osteoclast formation can be initiated. However, whether osteocytes are true “orchestrators of bone remodeling” cannot be verified from the animal models used to study osteocyte biology *in vivo*. Results from studying osteocyte biology using current animal models should come with the caveat that these models are not osteocyte-specific, and conclusions from these studies should be interpreted cautiously.

## 1 Introduction

Bone remodeling is an asynchronous cellular process signaling continuously and simultaneously throughout the skeleton, ensuring skeletal integrity and conferring functional adaptation. A basic multicellular unit (BMU) is the driver of bone remodeling and gives it its quantum character (1, 2). There is a distinct coupled role for each cell group in a BMU, and the balanced activity of each cell group ensures a balanced outcome at each bone remodeling site. Osteoclast resorbing activity is coupled with the recruitment and differentiation of mature bone-laying osteoblasts. In adulthood, this produces a neutral balance, i.e., the amount of bone excavated is replaced by just enough bone to fill in the space, leading to skeletal homeostasis. However, in pathology, the balance can be negative (bone is lost) or positive (bone is gained).

Cortical bone remodeling happens in osteons where blood vessels at the center of a Haversian canal supply the cells required to carry out bone remodeling. Remodeling in cortical bones occurs in the form of tunnels consisting of three zones, the cutting cone, the reversal zone, and the closing zone (3). The cutting cone consists of a majority surge of osteoclasts and their progenitors working to erode the bone surface. The reversal zone is a mingle zone of several cell types constituting what is known as reversal cells; these cells express markers of osteogenic commitment lineage, which intensifies more when the reversal zone comes closer to osteoid seams (4). Finally, the closing zone consists of a coordinated network of osteocytes which become embedded in the mineralizing bone matrix (3). Cancellous bone remodeling happens on the surface of trabeculae at the interface between the bone marrow and the bone surface in a trench-like structure rather than a tunnel (5). The BMU in cancellous bone consists of the same sequential appearance of osteoclasts, reversal cells including osteoblasts, and then embedding osteocytes.

Osteoblast differentiation proceeds from skeletal stem cells (6) to multiple early precursor stages before forming full-fledged matrix-secreting osteoblasts (7). Then, the cell encounters one of three possible fates: 60 to 80% of osteoblasts undergo apoptosis and the remaining osteoblasts become differentiated cells, either an osteocyte embedded in osteoid or a surface bone lining cell (8). The former (osteocytes embedding in the bone) represents a significant percentage of adult bone formation since 90% of bone cells are osteocytes (9, 10), and an average of 3.33 billion osteocytes are renewed every year through remodeling; that is approximately 7 to 10% of the total number of osteocytes (11).

Osteocytes have been termed the orchestrators of bone remodeling owing to the fact that they produce RANKL, which is obligatory for osteoclast formation and bone resorption, and they produce sclerostin which inhibits osteoblast formation and consequentlybone formation. Both osteoclast and osteoblast formation are necessary events for bone remodeling to take place, and they do take place in bones without osteocytes (anosteocytic bone). Owing to the property ascribed to osteocytes as “master regulators of bone remodeling”, the observation of bone remodeling in anosteocytic bones presents us with a puzzling question: are osteocytes required for bone remodeling?

Answering this question demands a comprehensive knowledge of the osteocyte entity in the bone. Therefore, in this review, we introduce osteocytogenesis from osteoblasts. We introduce anosteocytic bone and its characteristics in terms of its cellular components and remodeling ability. We also describe the role of the osteocyte in osteoclastogenesis and bone resorption, which is the initial event that takes place in the process of bone remodeling.

## 2 Becoming an osteocyte

The transition from an osteoblast to an osteocyte is accompanied by distinct functional and morphological changes. An osteoblast on the surface of the bone laying down the osteoid slows down its motion concomitant with a change from a polygonal/cuboidal shape to a flat almond-shaped cell body. Cell volume decreases by 30% and the cell becomes surrounded by unmineralized collagen fibers with patches of calcification. This cell is now called an osteoid osteocyte and like its predecessor (osteoblast), contains a prominent granular endoplasmic reticulum, a well-developed Golgi apparatus, numerous free ribosomes, and many mitochondria (12–14). On the mineral-facing side (the pre-existing bone side), the podia form, which later extends towards mineralizing fronts in the process of arborization (15). At this stage, the cell is polarized, which is evident by the direction of its dendrites and the arrangement of organelles in the cytoplasm. The nucleus rests on the vascular side (facing the bone surface) and the cell body is oriented with the long axis parallel to collagen fibers in the matrix (14, 16).

Once the cell is completely embedded in a mineralized matrix (termed young osteocyte and later mature osteocyte), cell volume becomes reduced by 70%. The embedded osteocyte experiences extensive arborization, and dendrites extend to the vascular surface [for a recent review of the control of dendrite formation, check Wang and Wein et al. (2022) (17)]. This gives osteocytes their distinctive star-shaped appearance (14). Osteocyte body size varies from 5 to 20 µm depending on the maturational stage, and their processes are branched and connected to osteocytes in the same plane and adjacent planes. The intracellular landscape of the mature osteocyte includes reduced Golgi apparatus and endoplasmic reticulum, and the cytoplasm/nucleus ratio decreases with prominently condensed chromatin and one or two nucleoli (14). This is paralleled by the cell gradually reducing its biosynthesis of the major extracellular matrix proteins, collagen type I, and other non-collagenous proteins that are considered osteoblast markers such as alkaline phosphatase, osteocalcin, and bone sialoprotein (18, 19).

The specification of an osteocytic transcriptional program leading to osteocyte differentiation from the IDG-SW3 cell line has been reported to be driven by glucose restriction, AMP-activated protein kinase, and proliferator-activated receptor γ co-activator-1 (PGC-1), which means that osteocytic transitioning from osteoblasts is accompanied by changes in cell bioenergetics that favor mitochondrial function. This signifies that osteocytes are programmed to accommodate an efficient energy production machinery due to their nutrient-restricted environment (20). Gene expression profiling of osteocytes and osteoblasts distinguishes proteins exclusive to osteocytes: E11/gp38, dentine matrix protein 1 (Dmp1), matrix extracellular phosphoglycoprotein (MEPE), phosphate-regulating endopeptidase homolog X-linked (PHEX), and sclerostin; of those, E11/gp38 is required for dendritic formation as its inhibition in the MLO-Y4 osteocytic cell line impedes dendritic formation (21).

The transition from an osteoblast to an osteocyte may give the impression that osteocytes are retired osteoblasts; however, osteocytes assuming this new architectural organization are dynamic cells that exhibit motility in the form of dendrite extension and retraction and their processes are rich in proteins involved in muscle contractile function (21, 22). After transitioning, osteocytes take on the form of an extensive network that is established by an active cleavage of bone proteins. During the embedding process, osteocyte processes “invade” the surrounding mineralized matrix by periosteocytic collagen cleavage that is dependent on membrane-type matrix metalloproteinase 1 (MT1-MMP) as MT1-MMP deficiency leads to osteocytes with significantly fewer and shorter processes that cannot reach out to communicate (23). This is also evident in mice that harbor a site-directed mutation in the pro α1(I) collagen gene (Col1a1^r/r^ mice). Col1a1^r/r^ mice are resistant to collagenase activity and their bones exhibit osteocytes with increased apoptosis and calvaria with a significantly higher number of empty lacunae (24).

Understanding the flow of events of osteocytogenesis is important because there is no one cut-off point at which a cell can be called a young or mature osteocyte. Suppose we are talking about an osteocyte that is embedded in an osteoid (osteoid osteocyte) and one that is embedded in a newly mineralized matrix (young osteocyte), and another buried further after many bouts of remodeling (old osteocyte), in such cases, we will be describing different osteocytes in morphological, transcriptional, and molecular profiles. Franz-Odendaal et al. (2006) suggested five schemes of differentiation, four of which describe how osteoblasts may become osteocytes, and the fifth describes how osteoblasts avoid getting trapped as is the case in anosteocytic bones of higher teleosts (discussed below) (25).

## 3 Specificity of murine models studying osteocytes *in vivo*

To study the function of osteocytes *in vivo*, researchers used the Dmp1 promoter (the 10-kb and the 8-kb) or the Sost promoter to drive recombinase activity and study the effect of gene knockdown in osteocytes or to drive the activity of reporter genes in osteocytes. However, debate arises concerning the specificity of these models.

Using the 10-kb Dmp1 promoter in R26 reporter mice showed that not only cells embedded in bone matrix express LacZ but also cells resting on newly formed bone matrix, i.e. osteoblasts or bone lining cells (26). Additionally, mice with 10-kb Dmp1-Cre-activated tdTomato expression (Ai9 reporter mice) showed positive tdTomato expression in osteocytes of cortical and cancellous regions, osteoblasts/bone lining cells, muscles, and bone marrow cells. The 8-kb Dmp1-Cre-activated tdTomato showed similar expression, but the degree of fluorescence intensity and the proportion of expression in cells resting on bone surfaces were less than that of the 10-kb Dmp1-driven Cre activity indicating a more specific but not osteocyte-discrete expression (27).

Osteocytes obtained through digestion and fluorescent activated cell sorting (FACS) of GFP fluorescent cells of (4–9) day-old pup calvaria under the control of the 8-kb Dmp1promoter revealed that GFP was also active in cells expressing dendritic cell-specific transmembrane protein (Dcstamp) and osteoclast-associated immunoglobulin-like receptor (Oscar), indicating that using the 8-kb Dmp1-Cre model drives the expression of GFP not only in osteocytes but also in cells of hematopoietic origin (0.33% of GFP positive cells were positive for markers of hematopoietic and endothelial cells). Hematopoietic cell depletion (Lin depletion) before GFP sorting eliminated the contamination and yielded a purer osteocyte population compared to sorting without hematopoietic depletion (28).

Osteoblast enriched population (double GFP/Lin negative) showed 2-fold higher RANKL and OPG expression than the highly pure osteocyte population (osteocytes with hematopoietic depletion) (28). Interestingly enough, osteocytes obtained with or without hemopoietic cell depletion could not support osteoclastogenesis and only supported the formation of TRAP+ cells with two nuclei but not three or more. Of note, a culture of (8-kb-Dmp1/GFP) osteocytes obtained from mouse calvaria (by fractionation or by fractionation and FACS) on plastic led to the loss of well-known osteocyte markers Dmp1, Sost, Mepe, and calcitonin receptor (Calcr) within 24 hours (28). Additionally, mRNA expression of Dmp1, Sost, and Calcr of osteocytes isolated by fractionation alone was lost after 24 hours of culture (29). Those observations may indicate that cells obtained using this preparation de-differentiated or died. This may provide some insight into why osteocytes obtained through this technique could not support osteoclast formation in culture (28).

To overcome the limitation of off-target recombination, another mouse model was developed under the control of regulatory elements of the Sost gene. Sost-Cre mice were crossed with tdTomato (red) Cre-reporter mice; the resulting mice did not show fluorescence in lining cells or osteoblasts. However, large numbers of red fluorescent cells were present in their bone marrow. Flow cytometric analysis of tdTomato-positive bone marrow cells revealed that Sost-Cre transgene is active in an early hematopoietic progenitor (30).

## 4 The lacunocanalicular network

The bone is hierarchically structured into cortical and cancellous bone, designed for optimal stress loading distribution, much like the design of construction bridge trusses. Within the cortical bone, there are three porosity levels. The first is the vascular porosity (osteon) represented by Haversian canals that contain capillaries and nerves and are mostly filled with interstitial fluid. The second and third represent the lacunae and canaliculi, respectively. Lacunae and canaliculi form the lacunocanalicular network spaced in concentric rings around one Haversian canal. The lacunae contain the body of the osteocyte and the canaliculi house osteocyte projections, which extend and form a network connecting osteocytes to other cells and the bone surface but do not cross osteonal cement lines (31).

The LCN is a vast transport and communication network, and it can be considered a specialty transport network that senses shifts in blood and interstitial molecule levels (such as phosphate, calcium, and nutrients) better than the vascular network alone, providing it with the function of quickly achieving homeostatic concentrations at the bodily level. In appreciating the vastness of the LCN, comparisons were made with other organs in the body where the surface area represents a functional rather than just a morphological character; for example, the total surface area of the LCN is larger than the skin, the gastrointestinal tract, and the adult lung (9). Within the local environment of the bone, osteocytes within the LCN cover a 100 times larger surface area than osteoblasts and osteoclasts cover the bone surface (11).

High-resolution imaging technologies reveal that the LCN density and geometry vary considerably even within the same singular bone (32). A synchrotron X-ray phase nano-tomography (SR-PNT)-based analysis of the LCN geometry revealed that lacunae are generally ellipsoidal while canaliculi are cylindrical. Lacunar shape correlated to their location within the osteon; inner lacunae are elongated, and outer border lacunae at the cement lines are flat spheroids. The canaliculi are spatially well arranged and uniformly distributed through the extracellular matrix, and their number correlates well with lacunar size, hence osteocyte size. Additionally, the canalicular volume is three times smaller than the lacunar pore volume, but the canalicular surface area is more than three times larger than the lacunae (33).

Buenzeli and Sims (2015) quantified osteocytes and their dendritic processes in the adult human skeleton using published literature obtained by modern imaging techniques (11). They estimated the average total number of lacunae to be 44 billion and live osteocytes to be 42 billion, indicating that approximately 1% of the osteocyte population is dead. Osteocytic processes were at 3.7 trillion processes with a total length of 175,000 km, 4.2 mm per single osteocyte, and 47µm per single cell process, forming 12.7 termini and up to 1128 termini per osteocyte and a total of 23.4 trillion connections, considering that these calculations are made for processes covering a cumulative length of 47 µm from the cell body. Those numbers are difficult to imagine, but the purpose of listing them here is to give an approximate mental image of the extreme connectedness and the labyrinthian hierarchy of the LCN that allows it to cover a total surface area of more than 201 m² at a small volume of approximately 17,500 mm³. This large surface area, with a relatively small volume of extracellular fluid running through the LCN, provides evidence of the highly efficient process by which osteocytes exchange information with their surroundings. The efficiency of the LCN as a transport network is essential given the fact that mineralized bone is impermeable to solutes, and the LCN is responsible for solute transport to deliver nutrients (i.e., information) and dispose of waste (34, 35).

The total number, length, and terminal connections of osteocytes are comparable to the elaborate neuronal connections, and both networks share molecular and behavioral similarities, especially with aging (36, 37). Additionally, the osteocyte transcriptome signature proposed by Youlten et al. is enriched in terms associated with axonogenesis, axon guidance, and development, which is not surprising, given that the most prominent feature of osteocytes is their dendrites (38). The LCN can transmit extensive information *via* direct contact and paracrine and endocrine communications at the whole organism level (10). As an organ’s complexity and size are associated with its functions and demands, Buenzeli and Sims’ work highlighted the magnitude of the effect that can be exerted by the totality of the osteocyte network in a single regulatory role (for example, in promoting osteoclast formation).

## 5 Osteoclastogenesis in anosteocytic bone

One paradoxical way of appreciating the role of osteocytes in the bone is studying bone devoid of osteocytes; ancestral aquatic bone is categorized into two types, cellular and acellular, and bone in some vertebrate groups (for example, modern teleosts) completely lack osteocytes (39, 40). Although flawed, the “acellular” terminology refers to bones devoid of osteocyte lacunae found in extant teleosts, a group representing half vertebrates and bony fish. Despite this terminology, the acellular bone contains cells (osteoblasts and osteoclasts) with functional similarity to those in the cellular bone, except for one cellular component that distinguishes the two types: the absence of osteocytes and their lacunae. The term anosteocytic bone was later coined by Weiss and Watabe in 1979, which referred to bone devoid of osteocytes (41). In this review, anosteocytic and acellular bone is going to be used interchangeably.

Two hypotheses were devised to explain the anosteocytic bone phenomenon. (1) Osteocytes are present in acellular bone, but they die, and their lacunae disappear by mineralization. However, this theory was revoked for lacking tangible evidence (39, 42), but was revisited again with circumferential evidence (43). (2) Osteoblasts never get incorporated in their secreted matrix and recede from the mineralization front and never become trapped as osteocytes (25, 44). In the latter scenario, osteoblasts are polarized and move away from a single osteogenic front as one unit laying down bone matrix synchronically, resulting in the formation of acellular bone (45, 46).

Studying the differences between bone types revealed that anosteocytic bone has lower stiffness but increased toughness compared to cellular bone (47–49) (**Table 1**). Previous light and electron microscopic images showed that both bone types contain a heterogenous cell population with similar cell zoning between cellular and anosteocytic bone, with the only difference being the absence of osteocytes. In terms of function, both types contain osteoblasts that lay the bone matrix and osteoclasts that resorb the bone matrix; however, osteoclasts in fish (both osteocytic and anosteocytic) are structurally different in that they lack ruffled borders and are mononucleated which rendered them undetected for some time (41, 51). Anosteocytic bone can remodel and undergo resorption in response to mechanical stress to a similar degree to that of cellular bone. One study compared descaling fish (a form of demineralization or resorption induction) of both bone types to signify the role of osteocytic osteolysis on resorption in descaled fish. In osteocytic bone, osteocytes had prominent perilacunar spaces indicating resorptive activity in the form of osteocytic osteolysis. Two types of osteoclasts were described, termed osteoclasts A and B; they were absent of ruffled borders with extensive endoplasmic reticula, indicating metabolic activity. Similarly, demineralized anosteocytic bone contained the same two types of osteoclasts, A and B, with similar structures to those of cellular bone but indicating that osteocytes were not needed to initiate osteoclast formation (41).

**Table 1:** The summary of differences between osteocytic and anosteocytic bone.

Character	Osteocytic bone	Anosteocytic bone	Reference
Microscopic gross structure	Hierarchical Haversian systems circled by secondary osteons.	Featureless at low magnifications and lamellated at higher magnifications with tubules traversing the lamella. (Specimen: Tilapia) Clearly demarcated overlapped hypermineralized remodeling rings and higher osteonal density (Specimen: Billed fish).	(47, 50)
Mechanical properties	Higher stiffness measured by nanoindentation and three-point bending tests (Human)	Higher toughness or more compliant (Tilapia)	(47, 49)
Porosity levels	Connected Haversian canals LCN Nanoporosities for water (between the collagen and the mineral and around the fibrils)	Lower porosity than osteocytic bone with tubular structures packed with collagen. Surface osteoblastic processes occupying canals of Williamson Nano porosities for water (Tilapia) Central cavity delineated by remodeling lines and off-center transverse vascular porosities with higher porosity than osteocytic bone (Billed fish)	(49, 50)
Remodeling potential	Modeling and remodeling occur throughout life.	Increases in load demand (water currents and hard diets) during intensive growth periods. Intense remodeling in billed fish (such as blue marlin) resembling secondary osteons.	(49, 50)
Osteoclasts	Mononucleated devoid of ruffled borders (osteocytic fish) Multinucleated with ruffled borders (mammals)	Mononucleated and devoid of ruffled borders	(41)
Osteoblasts	Present	Present	(41)

The dictum that osteocyte’s primary function is to orchestrate bone remodeling was challenged by demonstrating that anosteocytic bone of billfish rostra with heavy and cyclical cantilever loading exhibited heavily remodeled bone in the form of overlapping secondary osteons with “strike resemblance” to mammalian secondary osteons. The differentiating features were the absence of osteocytes and the higher spatial density of osteons (50). Also, anosteocytic osteons were an order of magnitude smaller than osteocytic osteons, rendering bone twice as much porous as osteocytic bone (50). Those features contribute to anosteocytic bone having an interconnected bone network that is similar to osteocytic (50) bone and can explain how anosteocytic bone of billfish can exhibit heavy remodeling since the bone surface can be readily accessed by osteoclasts for resorption. Nonetheless, the absence of osteocytes is rather perplexing since modern osteocytes are thought to respond to multiple redundant ways to initiate osteoclast formation and are major regulators of osteoclastogenesis and bone resorption. Osteocyte regulatory role in osteoclast formation (discussed below) is undeniable but may be dispensable for osteoclast formation and bone resorption.

Furthermore, acellular bone is less efficient in mineral homeostasis than osteocytic bone, partly due to the absence of osteocytic osteolysis. Osteocytic osteolysis is present in certain fish species that undergo periods of increased metabolic demand, such as in seasonal migration and during reproduction (52–54). This may indicate that mineral homeostasis is the evolutionary pressure favoring osteocytic bone, giving ancestral osteocytes the primary function of mineral homeostasis. Indeed, comparative histological data and phylogenetic distribution of both bone types highlight the role of osteocytes in mineral homeostasis through osteocytic osteolysis, which may be the primary reason for osteocyte reemergence [for a full review of phylogeny, refer to (39)].

The evolution of vertebrate endoskeleton is a milestone that conferred its species a survival advantage in the form of protection of neural tissues and vital organs, locomotion, and mineral homeostasis derived from its ground substance, calcium phosphate hydroxyapatite. Phosphorus is particularly critical for several reasons, one of which is that the most basic form of energy is a high phosphate molecule, the adenosine triphosphate (ATP). ATP utilization in aerobic glycolysis is the main pathway for energy bursts in vertebrates that would be impossible without ATP (55). In this respect, osteocytes are one of the primary regulators of systemic phosphate levels through Dmp1 and fibroblast growth factor 23 (FGF23); as in Dmp1 knockout osteocytes, FGF23 is released into the circulation, causing hypophosphatemia (56).

Mineral homeostasis is particularly important in terrestrial animals who live on land since aquatic ancestors (with cellular or acellular bone) can mobilize calcium and phosphorus from their surrounding environment through their digestive tract and gills; hence, bone is less critically active in mineral homeostasis than that of terrestrial animals in which osteocytes are majorly involved in its coordination.

In the study of fossilized animals, osteocytes can tell us a lot since osteocytes lacuna retain their shape in fossilized bone specimens as molds, and their structure (size and shape) is a proxy for the metabolic and mineral homeostatic ability of the studied animal. Using focused ion beam-scanning electron microscopy (FIB-SEM), researchers studied two taxa, osteostracans, and placoderms, of the earliest jawless and jawed vertebrates. Osteocyte lacunae in both taxa were comparable with their modern counterparts. The authors noted an area of low density contrasting the air-filled lacuna, referred to as an area of osteocytic osteolysis. This was indirect evidence of mineral metabolism of the first osteocytes (57).

## 6 RANKL expression by osteocytes

The role of osteocytes in altering osteoclast and osteoblast formation, thus, orchestrating bone remodeling, is known through the release of the receptor activator of nuclear factor κB ligand (RANKL) and sclerostin, respectively (58). RANKL is an obligatory cytokine that works with macrophage colony-stimulating factor (M-CSF) and pro-inflammatory cytokines to induce and maintain osteoclast formation and bone resorption (59, 60). Sclerostin is an inhibitor of Wnt coreceptors low-density lipoprotein receptor-related protein (LRP)5 and LRP6, antagonizing the canonical Wnt/β-catenin pathway that regulates the bone mass acquisition and osteoblast differentiation (61, 62).

Osteoclasts develop from hematopoietic monocytes when exposed to the myeloid and B-lymphoid specific transcription factor PU.1, which induces the expression of c-fms, the receptor for M-CSF (63). The now committed monocytes express RANK, which binds RANKL that is required to differentiate and activate osteoclasts. RANKL osteoclastogenic signaling can be neutralized by two receptors, osteoprotegerin (OPG) and leucine-rich repeat-containing G protein-coupled receptor (LRG4). OPG is a decoy-soluble RANKL receptor produced in many instances by the same cell expressing RANKL and prevents RANKL-RANK interaction. LRG4 competes with RANK and is found on the surface of osteoclast precursors and inhibits the nuclear factor of activated T cells (NFATC1) that initiates the necessary intracellular signaling cascade for osteoclast maturation and differentiation (64).

Different sources of RANKL contribute to bone remodeling and regardless of the source of RANKL, it is an obligatory osteoclastogenic factor that without osteoclast formation comes to a halt at varying degrees. Conditional deletion of RANKL in genetically distinct cell populations results in varying degrees of osteopetrosis. The deletion of RANKL from hypertrophic chondrocytes contributes to severe osteopetrosis and the presence of mineralized cartilage that is typically resorbed during the process of endochondral ossification due to the complete absence of osteoclasts (26). Conditional deletion of RANKL using osterix-Cre (active in late proliferating chondrocytes and early differentiating osteoblasts) or osteocalcin-Cre (active in mature osteoblasts) produced transgenic mice with defective tooth eruption, severe osteopetrosis, and unresorbed calcified cartilage. Conditional deletion of RANKL using Prx1-Cre mice resulted in osteoclast absence, higher bone mineral density, and severe osteopetrosis in the femurs. Additionally, the mice exhibited arrested tooth eruption, unresorbed cartilage, and widened growth plates in the distal femur, similar to RANKL or RANK germline deletion. Using Osx1-Cre mice led to a phenotype similar to that of Prx1-Cre mice. Ocn-Cre mice exhibited similar characteristics to the previous Cre models, except that some tooth eruption was observed in Ocn-Cre transgenic deletion (26).

Deletion of RANKL by targeting Cre activity under the control of the 10-kb Dmp1 promotor produced mice that were normal at 5 weeks of age and with no skeletal phenotype at birth, but which became progressively apparent with age. Dmp1-Cre mice (from 8 weeks) exhibited higher bone mineral density and cancellous bone mass in the femur and L4 vertebrae, and there was a significant (70%) decrease in the genomic RANKL content of collagenase-digested, osteocyte-enriched femoral cortical bones but only a modest (40%) decrease in whole tibias and L4 vertebrae (26, 65). This means that the contribution of RANKL produced by Dmp1-expressing cells is modest compared to the entire RANKL-expressing cell populations. Although only a modest decrease in RANKL expression was measured, this decrease led to a significant decrease in the number of osteoclasts in the cancellous bone of the distal femur of 6-month-old mice; additionally, the plasma of these mice had lower CTX and Ocn, while soluble RANKL did not change (26). As discussed above, it is important to note that since the 10-kb Dmp1 promoter is not osteocyte specific, this change may have been the result of the deletion of RANKL from subsets of osteoblasts and lining cells.

Deletion of RANKL under the regulatory elements of the Sost gene resulted in recombination in osteocytes but not in osteoblasts or lining cells. Sost-Cre mice had a higher cancellous bone mass and higher bone mineral density starting at 5 weeks of age. This phenotype increased at 22 weeks of age. Also, it resulted in a substantial decrease in RANKL genomic DNA in collagenase-digested femur shafts but not whole tibias and a significant decrease in RANKL mRNA expression in tibias, L5 vertebrae, and calvaria. Osteoclast number and surface area were significantly decreased in endocortical surfaces but increased more in cancellous regions. Those characteristics are similar to the skeletal phenotype that was generated using the 10-kb Dmp1 promoter (30).

Since the Sost-Cre transgene does not lead to recombination in lining cells or osteoblasts compared to the 10-kb Dmp1-Cre transgene (30), the Sost-Cre expressing cells (osteocytes in bone) signifies the role of osteocytes in supplying RANKL required for osteoclast formation in the context of physiological bone remodeling, but it does not mean that osteocytes are solely or exclusively responsible for supplying RANKL or supporting osteoclastogenesis. It means that they are part of the RANKL-producing cell populations and more so with increasing age. This is consolidated by the osteocyte transcriptome signature proposed by Youlten et al. (2022), which did not include (*TNFSF11*) the gene encoding RANKL in the signature transcriptome (38).

RANKL deletion driven by Sost-Cre showed a substantial decrease in osteoclast surface area per total bone area in the cancellous bone as opposed to endocortical regions (8 to 10% decrease *vs* 1 to 2%, respectively) (30), and given that 80% of remodeling happens in cancellous bone during growth, deletion of osteocytic RANKL could be recognized as an important source of RANKL in the vicinity of cancellous bone for bone remodeling during growth (66).

The sheer surface area of the osteocyte network makes it well suited to sense the need for initiating osteoclastogenesis and then act upon it. Given that osteocytes are embedded in the bone matrix, and the membrane-bound form of RANKL is the physiologically relevant form that is required for osteoclast formation, a question arises on how influential osteocyte membrane-bound RANKL is in the process of osteoclastogenesis. Research has demonstrated that osteocyte dendrites can contact cells on the bone surface and the bone marrow space (8, 11, 67). Since membrane-bound RANKL but not soluble RANKL supports osteoclastogenesis, it is of particular interest to show the contact between osteocytes and osteoclast precursors in *in vivo* animal models.

## 7 Osteoclastogenesis by osteocytes

Although it has become common wisdom that osteocytes directly initiate osteoclast formation through RANKL, osteoclastogenesis may also be initiated indirectly by other means (**Table 2**). Targeted ablation of osteocytes using the diphtheria toxin under the direction of the 10-kb Dmp1 promoter in mice with a constitutively active PTH/PTH-related peptide (PTHrP) type 1 receptor (PTH1R) in osteocytes increased osteoclast number and bone resorption (77, 86). Additionally, PTH1R signaling induces RANKL expression, and its deletion in osteocytes using the 10-kb Dmp1 promoter to drive recombinase activity abrogates osteocytic osteoclastogenic potential and RANKL expression (87).

**Table 2:** The high degree of redundancy built in the osteocytic-driven bone resorption and stimuli impact on effector cells including osteoclasts, osteoblasts, and osteocytes themselves.

Stimulus	Effect on osteocyte	Effect on cellular remodeling	Reference
Inflammatory cytokines (TNF-α, IL-1β, IL-6)	Expression of RANKL (*TNFSF11*) and Sclerostin (*SOST*)	Promotes osteoclastogenesis and halts osteoblast differentiation	(68–71) (72)
Apoptosis by Sex steroid deficiency Fatigue loading Weightlessness Glucocorticoids Aging	Expression of RANKL and Sclerostin by neighboring osteocytes	Osteoclast recruitment and differentiation. Halts osteoblast differentiation.	(73) (74, 75) (64) (30) (76)
(PTH/PTHr) signaling	Expression of RANKL Inhibition of Sclerostin	Increases osteoclastogenesis and reduces osteoblast apoptosis	(77)
Phosphate homeostasis	Release of FGF 23	Biphasic: Partially inhibits early differentiation Augments osteoclast activity at low concentrations	(78)
Necrosis	Release of DAMPs (small nuclear ribonucleoprotein SAP -130)	Osteoclast recruitment and differentiation	(79)
Information trafficking loss by Cx 43 deletion	Expression of RANKL and HMGB 1	Osteoclast recruitment and differentiation	(80, 81)
Senescence	Acquiring SASP Expression of RANKL Reduced Wnt 16 and OPG (*TNFRSF11B*)		(82, 83)
Lactation and rapid release of calcium Parathyroid hormone-related peptide (PTHrP)	Release of Cathepsin K (*CTSK*), tartrate acid phosphatase (*Acp5*), Matrix metalloproteinase 13 (MMP13), carbonic anhydrase 1, 2, and vacuole ATPase components	Induces osteocytic perilacunar remolding	(84, 85)

**Figure 1:**
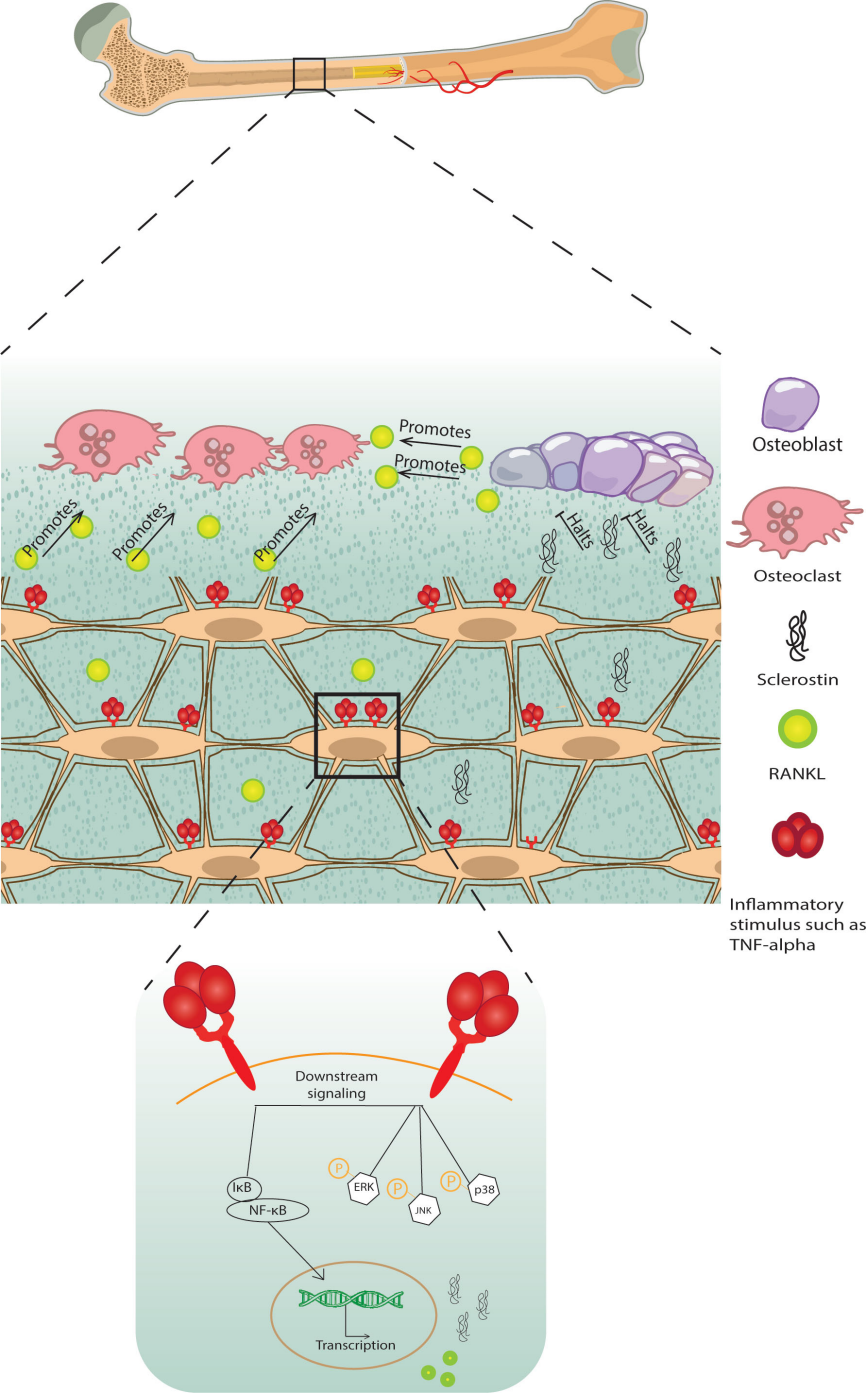
A schematic diagram of bone loss due to inflammatory stimuli cytokine influx. Inflammatory stimuli such as TNF-α and IL-1β lead to osteocyte and osteoblast RANKL expression and osteocyte sclerostin expression, which induces osteoclastogenesis and halts osteoblast formation, respectively, negatively modulating bone remodeling.

Pro-inflammatory signals such as TNF-α, IL-1β, IL-6, and IL-17, act on osteocytes to increase their RANKL expression, osteoclastogenesis, and bone remodeling or to increase sclerostin expression which contributes to reduced bone formation (**Figure 1**) (68–71) [for a comprehensive review of osteocyte secreted factors regulating osteoclastogenesis, check (88)]. TNF-α is a pleiotropic cytokine with a well-characterized pro-inflammatory effect; it is elevated in chronic inflammatory diseases including but not limited to obesity, diabetes, osteolytic bone diseases such as rheumatoid arthritis, and infectious inflammatory conditions such as periodontitis (89). Osteocytes express both TNF-α receptors TNFR I and II and signaling through TNFRs induced MAPKs phosphorylation and the NF-κB pathway activation to increase osteocyte RANKL and sclerostin expressions to initiate osteoclast formation and reduce osteoblast differentiation *in vitro* and *in vivo* (68, 72). Osteocytes may also control osteoclast formation independently of RANKL through FGF23. *In vitro* early-stage cultures of human monocytes with M-CSF and RANKL exhibited decreased osteoclastogenesis when supplemented with FGF 23, and this effect was abolished with the addition of a pan FGF23 inhibitor (78).

Osteocyte apoptosis is accompanied by bone resorption, another mode of action through which osteocytes recruit osteoclasts either by signaling neighboring cells to express RANKL or independent of RANKL by releasing apoptotic bodies, which recruit osteoclast precursors (73, 90). Additionally, apoptotic osteocytes undergo secondary necrosis due to their position in bone, which prevents their phagocytosis; this is accompanied by the release of damage-associated molecular patterns (DAMPs), which activate the monocytic release of inflammatory cytokines that amplify osteoclastogenic signals and lead to the release of RANKL in osteoblast and stromal cells (91). Of note, this mode of osteoclast recruitment and differentiation is not supported by osteoblast apoptosis or osteoblastic apoptotic bodies (90). Apoptosis of osteocytes is seen in glucocorticoid treatment and sex steroid deficiency, such as in ovariectomized rats and mice, and suppressing osteocyte apoptosis induced by sex steroid deficiency inhibits bone resorption (73, 92).

Mechanical signals conveyed to osteocytes modulate osteocyte apoptosis and osteocytes near microcracks resulting from fatigue loading the bone are apoptotic osteocytes that release ATP and signal to bystander osteocytes to express RANKL, which recruits osteoclasts to initiate bone resorption and remodeling (74, 75, 93). Contrastingly, complete weightlessness in unloading leads to osteocyte apoptosis which signals osteoclast recruitment and bone resorption (26, 94). Inducing osteocyte necrosis *via* the diphtheria toxin recruits osteoclasts, increases their numbers, and produces an osteoporotic phenotype. Necrotic osteocytes release DAMPs, which act as signals for pattern recognition receptors, specifically C-type lectin receptor Mincle on the surface of macrophages which triggers recruitment and differentiation to osteoclasts (79).

Aging osteocytes but not osteoblasts are noted to have a senescent character with DNA damage, mitochondrial dysfunction, telomere dysfunction-induced foci (TIFs), and senescence-associated distension of satellite DNA (SADS) (95). Senescent osteocytes acquire a distinct secretome termed senescent-associated secretory phenotype (SASP), increased RANKL expression, and decreased Wnt16 expression, which is known to inhibit osteoclastogenesis by inducing OPG expression (76, 82, 83, 96).

Administering diphtheria toxin to mice harboring a diphtheria toxin receptor guided by the 10-kb Dmp1 promoter resulted in osteocyte-less mice (OL). OL mice were not completely absent of osteocytes. At 18 weeks of age, the mice exhibited a comparable LCN fluid space, but the connectivity of the osteocyte network was markedly decreased, and the cells had a decreased nuclear size; therefore, the authors termed the mice osteocyte-less where some osteocytes were killed and ablated while others were compromised. RANKL mRNA and the number of osteoclasts were slightly elevated in OL mice; bone remodeling rate as assessed by calcein double labeling did not differ between WT and OL mice (97). The increase in RANKL mRNA and consequently osteoclast numbers in the bone of OL mice may have been due to the fact that ablated (dead) osteocytes signal to the neighboring cells that survived the ablation to release RANKL; however, this hypothesis was not tested.

In another study, the 10-kb Dmp1 promoter was used to ablate osteocytes using diphtheria toxin which caused the mice to have severe sarcopenia, osteoporosis, and degenerative kyphosis at the early stage of 13 weeks, leading to a shorter lifespan. Ablation of osteocytes in these mice caused impairment of osteogenesis and lymphopoiesis, and increased cellular senescence of total bone marrow, primary spongiosa, and cortical bone. It also increased osteoclastogenesis and expanded myeloid lineage differentiation (98).

The transgenic mice at E19.0 had no apparent skeletal phenotype indicating that embryonic development of skeletal tissue appeared to be normal. However, at 4 weeks, transgenic mice had more empty lacunae (60%) devoid of osteocytes within the cortical and cancellous bone matrix. The empty lacunae number increased at week 13 to 80%, which means that the diphtheria toxin had an accumulative effect with age. Mice at 4 weeks exhibited cancellous and cortical bone loss, which was exacerbated with age (at 13 weeks of age). At 13 weeks of age, the mice developed kyphosis due to retardation of vertebral maturation accompanied by vertebral osteoporosis, which led to the development of gait abnormalities. The mice exhibited body weight and muscle tissue loss and focal atrophy shown by examination of gastrocnemius muscle. The mice exhibited reduced osteocalcin gene expression, increased RANKL, and increased RANKL/OPG ratio at 4 weeks. Additionally, histomorphometry analysis revealed that osteoclast surface numbers (N.Oc/BS) significantly increased after osteocytes ablation, and serum collagen type I c-telopeptide (CTX), a bone resorption index, was also significantly increased, which indicated a high level of osteoclast activity (98).

However, knowing that the 10-kb Dmp1 promoter (as discussed above) is not osteocyte specific, these results should be interpreted with caution and studied in comparison to other Cre mouse models that drive recombination in cells of the bone niche since the possibility that other cell populations contributed to the developed phenotype stands still.

## 8 Discussion

Osteocytes have been termed master manipulators of bone remodeling. But the fact that bones that are devoid of osteocytes experience bone remodeling raises the question of how important osteocytes are for this role. In this review, we discuss osteocytogenesis and the formation of the LCN to showcase the complexity of the osteocyte entity. We discuss current Cre murine models used for the study of osteocyte osteoclastogenic potential through RANKL deletion and highlight their limitations. The importance of understanding osteocyte transitioning from osteoblasts lies in the notion that osteocyte differentiation follows a spectrum. The current murine models used to study osteocyte biology *in vivo* are not specific to osteocytes and drive the expression of mutations or ablations in osteoblasts, lining cells, and cells residing in the bone marrow. Studies using the Dmp1-Cre or the Sost-Cre to drive RANKL deletion have been shown to drive recombination in other cell types, as discussed above, which calls into question the size of the contribution of osteocytes in supplying RANKL required for osteoclastogenesis. The results of these studies are certainly not to be discarded, but they should be critically analyzed and compared to other Cre models targeting other cell populations within the bone niche, which has provided us with a greater understanding of osteocyte biology and their osteoclastogenic potential (99). Additionally, the observation that those models experienced said phenotypes with increasing age was consistent among the mouse models used to study osteocytes which gives credence to the likelihood of their specificity.

Osteocyte apoptosis and deterioration of the LCN are associated with increased osteoclastogenesis and contribute to bone loss; whether osteocyte apoptosis is causative was highlighted in *in vitro* studies using osteocyte apoptotic bodies to drive osteoclast formation, but rigorous *in vivo* studies using a specific osteocyte marker to drive recombination are required. Additionally, studies on bone devoid of osteocytes show that osteocytes are not necessary for osteoclast formation, as bone devoid of osteocytes undergoes resorption and remodeling. Since RANKL can be supplied from other cell types in anosteocytic bone, this means that osteocytes are not essential for this role in anosteocytic bone but contribute to it in osteocytic bone.

Paracrine and endocrine signals act on osteocytes and in turn, osteocytes relay these signals and translate them to effector molecules that control bone remodeling (99). Osteocyte molecules are a target for therapeutic discoveries not only in the physiological regulation of bone remodeling but also in the regulation of remodeling as a complication of conditions such as diabetes and cancer and as endocrine factors that affect phosphate balance, glucose metabolism, and fat mass [for a comprehensive review, check (99)]. Among the most well-characterized molecules are RANKL and sclerostin. Therapies targeting those molecules have demonstrated their success, highlighting the role of the osteocyte not only in physiological bone remodeling but also in pathological bone loss. Our understanding of osteocytes has advanced tremendously; they are regulators of bone remodeling and endocrine cells that affect distant organs; the question of how anosteocytic bone achieves the feat of bone remodeling of osteocytic bones raises the possibility of the presence of other mechanisms regulating bone remodeling independent of osteocytes. Answering this question will broaden our understanding of bone biology and the remodeling of bone beyond the osteocyte.

## Author contributions

AM and HK contributed to the conceptualization, supervision, and original draft preparation. AM, HK, and IM contributed to the critical revision of the manuscript. FO and TN contributed to writing, reviewing, and editing. All authors contributed to the article and approved the submitted version.
